# Improved saccharification of steam-exploded *Pinus radiata* on supplementing crude extract of *Penicillium* sp.

**DOI:** 10.1007/s13205-014-0212-2

**Published:** 2014-04-06

**Authors:** Hamish Cameron, Sylke H. Campion, Tripti Singh, Alankar A. Vaidya

**Affiliations:** 1 Faculty of Science and Engineering, University of Waikato, Private Bag 3105, Hamilton, New Zealand; 2 Scion, Te Papa Tipu Innovation Park, Private Bag 3020, Rotorua, 3046 New Zealand

**Keywords:** Accessory enzyme, Fungi, Hemicellulase, β-Mannanase, Saccharification, Softwood

## Abstract

Commercially available enzymes do not contain all the necessary softwood-specific accessory enzymes to obtain high saccharification efficiency. In this work, six saprophytic fungi obtained from *Pinus radiata* plantation site were screened for the putative softwood-specific accessory enzyme, β-mannanase. A *Penicillium* sp. was found to produce β-mannanase in both solid (31.6 units/g of dry biomass) and liquid (117 units/g of dry biomass) cultures using locust bean gum as an inducer after 2 weeks of incubation. The saccharification of steam-exploded *Pinus radiata* was 7.8 % w/w improved when the crude extract of *Penicillium* sp. was added to a mixture of commercial enzymes.

**Electronic supplementary material:**

The online version of this article (doi:10.1007/s13205-014-0212-2) contains supplementary material, which is available to authorized users.

## Introduction

Woody biomass can be used as a feed-stock for the production of sugars which can be converted to biofuels and bio-chemicals (Zhu and Pan 2010). Enzymes can be used to break down wood into sugars but this is an expensive step due to the cost of enzymes (Berlin et al. 2007). An alternative strategy is to decrease the cost of enzymes by producing a crude enzyme extract on-site, rather than using a commercial enzyme. Moreover, commercially available enzymes are limited in number and composition and have generally been optimized for non-woody herbaceous biomass (Banerjee et al. 2010; Berlin et al. 2007). In the case of herbaceous and hardwood plant species, the hemicellulose is xylan rich whereas for softwood it is mannan rich (Juturu and Wu 2013). In softwood, the presence of hemicellulose may limit the hydrolysis of cellulose in the absence of accessory enzymes (Várnai et al. 2011).

β-Mannanase (E.C. 3.2.1.78) is a key softwood-specific accessory enzyme as it cleaves the main chain of both glucomannans and galactoglucomannans (GGMs) into oligosaccharides (Várnai et al. 2011; Yang et al. 2009). Another strategy for obtaining a complete enzyme mixture involves blending crude enzyme extracts having softwood-specific accessory enzyme with commercial enzymes. This approach has great potential since no activities are lost and it can lead to enzyme synergy and better saccharification efficiency (Yang et al. 2009).

In the screening of fungi for specific enzymes capable of degrading softwood biomass, it is crucial to search in the ecological niche where the desired biomass is growing. Hence, in this work, saprophytic fungi that grow in *Pinus radiata* plantations were selected (Visser et al. 2013; Singh et al. 2008; Zhang and Lynd 2004). Two fungi, each from the categories of brown rot, soft rot and mould were selected. The effect of solid versus liquid medium and incubation time on the extracellular production of β-mannanase from these six species was studied. For the first time, improvement in saccharification of steam-exploded *Pinus radiata* substrate is demonstrated after supplementing commercial enzyme with crude enzyme preparation from New Zealand native *Penicillium* sp.

## Materials and methods

All fungi were obtained from Scion’s mycological depository. The commercial enzymes Celluclast 1.5L and Novo-188 were obtained from Novozymes A/S. All other chemicals were purchased from Sigma-Aldrich and were used as received. An individual culture of each fungus was transferred from their glycerol stocks (40 % w/v glycerol) aseptically onto 1.5 % w/v malt agar nutrient medium and incubated in the dark at 75 % relative humidity and 25 °C. Each fungus was grown for 2 weeks till mycelium and fungal colonies are clearly seen. These plates are used as the stock cultures in sub-culturing onto the fresh solid or liquid media.

### Growth on solid medium

Malt agar (0.5 % w/v) was prepared by dissolving 10 g of malt and 12 g of agar in 2 L deionized water with heating. While still liquid, it was divided equally into two 1 L flasks. One flask contained 5 g locust bean gum (LBG; 0.5 % w/v) and the other flask was kept as a control. The pH was adjusted to 5.0 using 0.4 % HCl or 0.5 M NaOH and both flasks were autoclaved at 121 °C for 20 min. The warm media were poured into pre-sterilized Petri-plates (40 mL per plate). The plates were allowed to cool to room temperature and then each plate was inoculated with 5-mm diameter fungal plugs punched from stock culture plates. Duplicate plates were prepared for each fungus.

### Growth in liquid media

Broth was prepared by dissolving, KH_2_PO_4_ 1 g, MgSO_4_ 0.5 g, ammonium tartrate 0.2 g, NaH_2_PO_4_ 0.2 g, CaCl_2_ 0.05 g, FeSO_4_ 0.05 g, CuSO_4_ 0.01 g, ZnSO_4_ 0.005 g, MnSO_4_ 0.005 g per litre of deionized water. Two litres of liquid medium was prepared and divided equally into two 1 L flasks. One flask contained 5 g locust bean gum (LBG; 0.5 % w/v) and the other flask was kept as control. In 100 mL capacity conical flasks, 40 mL of either control or locust bean gum (LBG) liquid medium was poured and flasks autoclaved at 121 °C for 20 min and then cooled to room temperature. Each flask was inoculated at room temperature using 5-mm diameter plugs punched from stock culture plates. Duplicate flasks were prepared for each fungus.

### Fungal growth and crude enzyme recovery

The inoculated samples were incubated in the dark at 25 °C and at 75 % relative humidity without shaking. At defined time intervals (7, 14, 21 and 28 days), duplicate samples of each fungus were removed and crude β-mannanase was recovered from solid medium as follows––at each time point, culture grown on solid medium was cut into 1 × 1 × 0.5 cm cubes and transferred into separate 50 mL capacity centrifuge (Falcon) tubes and crushed to fine particles. A 30 mL of 0.05 M Na-citrate buffer (pH 5) was added and the mixture was incubated in a rotatory shaker at 15 °C for 18 h at 220 rpm. Then, each sample was centrifuged at 10,000×*g* for 20 min. The supernatant was decanted and the precipitate was washed once with 10 mL of 0.05 M Na-citrate buffer pH 5 and centrifuged at 10,000×*g* for 20 min. The washing was mixed with the supernatant and kept in a refrigerator at 8 °C for further use. The solid mass precipitated was freeze dried and weighed. The weight of the dry biomass was calculated from the difference in dry weight of fungal culture grown on locust bean gum—solid medium and the control. Crude β-mannanase recovery from liquid medium—the contents from each flask were transferred to a 50 mL capacity centrifuge (Falcon) tube and centrifuged at 10,000×*g* for 20 min. The supernatant was decanted and kept in a refrigerator at 8 °C for further use. The precipitated biomass was freeze dried and weighed.

### Enzyme assays

**Fig. 1:**
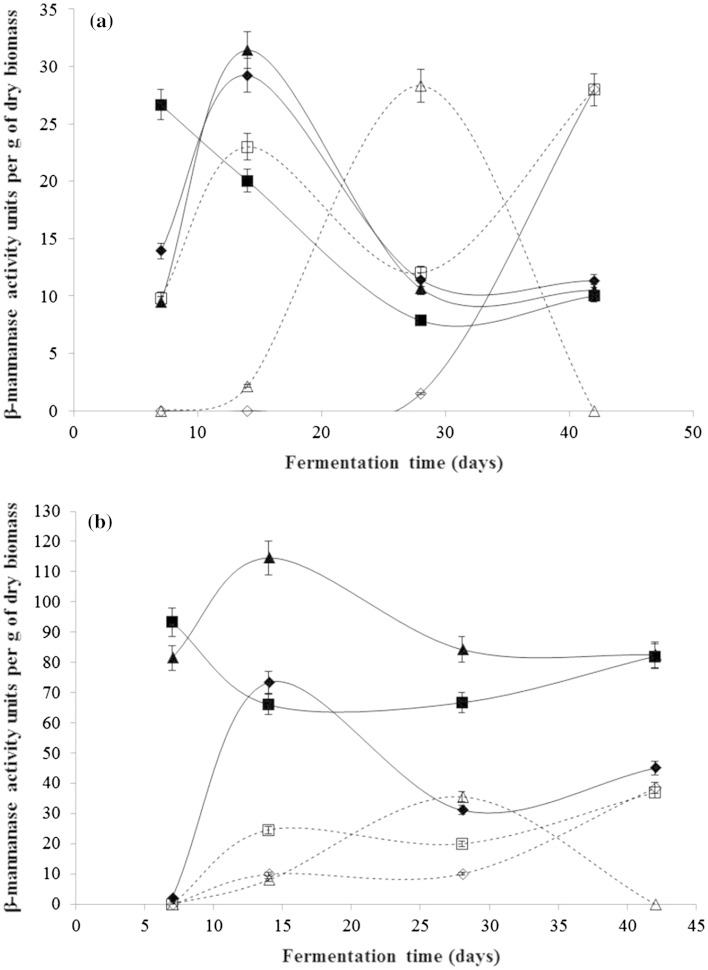
**a** Crude β-mannanase recovery from solid medium. **b** Crude β-mannanase recovery from liquid medium. Data shown here are average of quadruplicate measurements with corresponding relative standard deviations. *Filled trianglesPenicillium* sp., *empty trianglesCladosporumherbarum*, *filled squaresOligoporusplacenta*, *empty squaresLenzitestrabea*, *filled diamondsCephalosporium* sp. and *empty diamondsChaetomiumglobosum*

β-Mannanase activity in the supernatant was measured using a partially depolymerized Carob-galactomannan conjugated with Remazol brilliant blue R (Megazyme, Ireland) as the substrate. The substrate stock solution was prepared at 1 % w/v in 0.2 M sodium acetate buffer pH 5.0. The substrate stock and crude enzyme supernatant were incubated separately at 40 °C for 3 h before the assay was performed. In 5 mL capacity glass tubes, 0.5 mL of stock substrate solution was mixed with 0.5 mL of crude enzyme solution and incubated at 40 °C for 2.5 h. The reaction was terminated by the addition of 2.5 mL of ethanol (95 % v/v). After brief vortexing, the absorbance of supernatant was measured at 590 nm. Enzyme activity was determined from a standard calibration curve (*R*^2^ = 0.99) of Remazol brilliant blue R solution (0.008–0.00016 μM) prepared in 0.2 M sodium acetate buffer pH 5. One unit of β-mannanase activity was defined as the amount of enzyme which released 0.001 μM of dye under assay conditions. Each assay was performed in quadruplicate and the average value is presented in Fig. 1. β-Glucosidase (E.C. 3.2.1.21) and β-mannosidase (E.C. 3.2.1.25) activity were each measured using 10 mM of standard chromogenic substrates, i.e. *p*-nitrophenyl-β-d-glucoside and *p*-nitrophenyl-β-d-mannoside, respectively (Bailey and Nevalainen 1981). One unit of activity was defined as the amount of enzyme which released 1 μM of *p*-nitrophenol from their respective standard substrates under assay conditions.

### Enzymatic hydrolysis of steam exploded (SEW) *Pinus radiata*

SEW was produced in the steam explosion apparatus (Newman et al. 2013). 0.75 kg chips were taken with a moisture content of 60 %, impregnated with SO_2_ (3 % w/w) and heated with steam at 215 °C for 3 min. The SEW pulp was washed four times with water and oven dried at 110 °C to obtain a 54 % yield. This substrate was hydrolysed using commercial enzyme in the form of Celluclast 1.5L and Novo-188 at 10 FPU:12.5 CBU per g of substrate in a 5-mL saccharification mixture with and without addition of the freeze dried crude extract of *Penicillium* sp. at 100 mg/mL. A control experiment was run in parallel in which hydrolysis of SEW was performed using only crude extract of *Penicillium* sp. at 100 mg/mL in 5-mL mixture. All experiments were incubated at 180 rpm agitation speed in an inclined vibratory shaker for 72 h. Experiments were carried using a substrate concentration of 1.5 % on a dry basis. The sugar composition (monomeric and oligomeric) of the saccharified filtrate was determined using ion chromatography as described elsewhere (Vaidya and Singh 2012).

## Results and discussion

Cell growth for all six test fungi was normal when grown on control medium (without locust bean gum) but no β-mannanase activity was detected. This indicates that the presence of locust bean gum did not affect cell growth but did lead to extracellular secretion of enzymes required for the degradation of glucomannan. All fungi (except *Cladosporum herbarum* and *Lenzites trabea*) showed almost 200–400 % higher β-mannanase activity units per g of dry biomass when grown in liquid medium compared to solid medium (Fig. 1a, b). The improved enzyme production in liquid medium can be due to better accessibility of growing hyphae to locust bean gum.

Generally, the time profile of enzyme production is similar for a given fungi in solid and in liquid medium. An early induction period (7 days) was seen in the case of the brown rot *Oligoporus placenta* whereas *Lenzites trabea* only produced β-mannanase after a 14-day growth period and maximum activity occurred only after 42 days. Soft rot fungi had different induction responses to locust bean gum. *Cephalosporium* sp. produced the most activity at 14 days but then had an inconsistent response to locust bean gum, whereas *Chaetomium globosum* reached maximum production after 42 days. The two mould fungi *Penicillium* sp. and *Cladosporum herbarum* showed the highest β-mannanase activity at 14 and 28 days incubation period, respectively, and activity reduced thereafter.

Among six fungi tested, *Penicillium* sp. showed highest β-mannanase activity per g of dry biomass in both liquid and solid media. Moreover, it also showed low β-mannosidase activity (12.9 units) in accordance with the rare appearance of fungal β-mannosidase in the literature (Maijala et al. 2012; Valáŝkova and Baldrian 2006). Both β-mannanase and β-mannosidase activities are relevant to softwood glucomannan hydrolysis. Interestingly, none of the fungi expressed β-glucosidase activity either in liquid or solid medium.

Hydrolysis of SEW using Celluclast 1.5L:Novo-188 at 10 FPU:12.5 CBU per g of substrate gave 2.10 ± 0.1 g of total glycosyl units (monomeric + oligomeric) per litre, which correspond to 26.1 ± 1.3 % w/w of polysaccharide conversion (Table 1). No mannosyl or galactosyl units were released from SEW using a mixture of commercial enzymes only. This is to be expected because the commercial enzyme preparation Celluclast 1.5L contains α-l-arabinofuranosidase, endo-xylanase and β-xylosidase suitable for degrading hardwood hemicellulose but lacks mannan-degrading enzymes, such as β-mannanase and/or β-mannosidase, which act as accessory enzymes in the hydrolysis of glucomannan and GGMs in softwood hemicellulose (Juturu and Wu 2013). When the commercial enzyme was supplemented with the crude enzyme preparation from *Penicillium* sp. the production of monomeric glucosyl units increased from 1.85 ± 0.1 to 2.48 ± 0.1 g/L. This corresponds to a 7.8 ± 0.4 % w/w improvement in monomeric glucosyl released. This improvement in release of glucosyl units (g/L) during saccharification is due to the synergistic action of following enzymes––cellulase available from Celluclast 1.5L and crude extract of *Penicillium* sp. (for details refer to supplementary information), β-glucosidase available from Novo-188 and the presence of β-mannanase and β-mannosidase in the crude extract of *Penicillium* sp. Furthermore, from control experiment in which only crude extract of *Penicillium* sp. is used for SEW hydrolysis, a small amount of monomeric glucosyl units (0.10 g/L) released because of lack of β-glucosidase activity in the crude extract. This in turn resulted in an accumulation of oligomeric glucosyl units (0.41 g/L; 5 % w/w cellulose conversion). Only in presence of crude extract of *Penicillium* sp., the conversion of soluble polymeric mannan to monomeric and oligomeric mannosyl units is achieved albeit the original hemicellulose content in SEW is small (0.3 % w/w) (Table 1). To corroborate this result further a comparison of crude extract of *Penicillium* sp. against commercial enzymes (both were taken on equal protein basis of 10 mg/mL) in hydrolysis of locust bean gum (1 % w/w) showed that using only crude extract twofold enhanced activity was obtained compared to commercial enzyme.

**Table 1:** Saccharification of SEW

	Monomeric sugars g/L (% w/w conversion)			Oligomeric sugars g/L (% w/w conversion)		
	Glucose	Mannose	Galactose	Glucosyl units	Mannosyl units	Galactosyl units
Commercial enzyme	1.85 (23.0)^a^	0.00	0.00	0.25 (3.1)^a^	0.00	0.00
*Penicillium* sp. crude extract (control)	0.10 (1.24)^a^	0.01 (33.3)^a^	0.00	0.41 (5.08)^a^	0.02 (66.7)^a^	0.01 (66.7)^a^
Commercial enzyme + *Penicillium* sp. extract	2.48 (30.8)^a^	0.01 (33.3)^a^	0.00	0.00	0.02 (66.7)^a^	0.01 (66.7)^a^

All values in the table are the mean of triplicate experiments and have a standard deviation of ±5 %. The sugar composition (% w/w) of original SEW is––glucosyl units = 53.6; mannosyl units = 0.3; galactosyl units = 0.1

^a^The numbers shown in the parentheses are % w/w conversion of sugars from the original SEW sample taken in 5 mL saccharification

Thus, the crude preparation of *Penicillium* sp. has a boosting effect in the hydrolysis of pretreated softwood substrate and emphasizes the importance of a feedstock specific mixture of hemicellulase and cellulase enzymes (Gao et al. 2011). Várnai et al. (2011) reported a 4 % w/w increase in the hydrolysis of steam pretreated spruce when *Trichoderma reesei* β-mannanase supplemented at 1:4 w/w on protein basis with cellulases mixture (Várnai et al. 2011). This work indicates that the effectiveness of a mixture of commercial cellulases can be improved by adding biomass-specific accessory enzymes and, therefore, has commercial implications in the production of biofuel enzymes. Additional work on enzyme purification and improving production of β-mannanase by whole genome mutagenesis of *Penicillium* sp. or heterologous expression of the β-mannanase gene into *Pichia pastoris* are a subject matter of future communication.

## Electronic supplementary material

Below is the link to the electronic supplementary material.

Supplementary material 1 (DOCX 88 kb)
