# Supplementary material for: Improved saccharification of steam-exploded Pinus radiata on supplementing crude extract of Penicillium sp
Source: 3 Biotech. 2014 Apr 6;5(2):221–5. doi: 10.1007/s13205-014-0212-2 (PMC4362734; doi:10.1007/s13205-014-0212-2)
Supplement: Supplementary file 1 — Supplementary material 1 (DOCX 88 kb) [file 13205_2014_212_MOESM1_ESM.docx]

**Supplementary information**

The protein band of a well characterized pure β-mannanase from *Aspergillus niger,* a mould fungus (Figure A1; lane 3) matches with the protein band from the crude extract of *Penicillium* sp. (lane 2). Other protein bands were also present in the crude extract of *Penicillium* sp. which was expected considering no purification steps were performed. The other protein bands in the crude extract may originate from the multiplicity of hemicellulases or the presence of other proteins involved in cellulose hydrolysis. The presence of other proteins in addition to β-mannanase is likely to be beneficial for the degradation of complex woody materials.


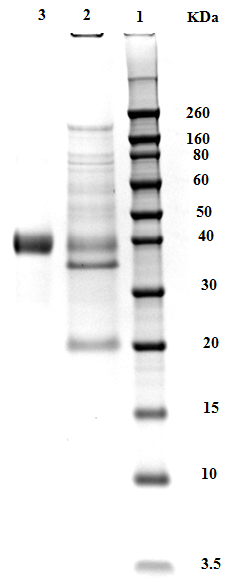


**Fig. A1** SDS-PAGE of crude enzyme preparation of *Penicillium* sp. grown on liquid medium for 2 weeks (lane 2), Protein molecular weight markers (lane 1), known pure β-mannanase from *Aspergillus niger* (lane 3). SDS-PAGE was carried out on 12 % polyacrylamide gels as described by Laemmli (1970) (Laemmli 1970).

**Reference**

Laemmli UK (1970) Cleavage of structural proteins during the assembly of the head of bacteriophage T- lambda. Nature 227:680-685
